# Melatonin Improves Bovine Embryo Production and Quality via Antioxidant, Metabolic, and Epigenetic Pathways

**DOI:** 10.3390/antiox14111322

**Published:** 2025-11-01

**Authors:** Hallya Beatriz Sousa Amaral, Márcia Marques Silveira, Ana Caroline Chaves Vall Nicolás, Laryssa Ketelyn Lima Pimenta, José Eduardo Vieira Chaves, Alexandre Rodrigues Caetano, Maurício Machaim Franco, Margot Alves Nunes Dode

**Affiliations:** 1 Faculty of Agronomy and Veterinary Medicine, University of Brasilia, Brasilia 70.910-900, DF, Brazil; sousahallya@gmail.com (H.B.S.A.); carolnicolas015@gmail.com (A.C.C.V.N.); 2 Federal District Research Support Foundation—FAP DF, Brasilia 70.636-000, DF, Brazil; marciamarquessilveira@gmail.com; 3 Embrapa Genetic Resources and Biotechnology, Brasilia 70.770-901, DF, Brazil; alexandre.caetano@embrapa.br (A.R.C.); mauricio.franco@embrapa.br (M.M.F.); 4 Institute of Biological Sciences, University of Brasilia, Brasilia 70.910-900, DF, Brazil; laryketelyn22@gmail.com; 5 School of Veterinary Medicine and Animal Science, Federal University of Goiás—UFG, Goiânia 74.001-970, GO, Brazil; josechaves@discente.ufg.br; 6 School of Veterinary Medicine, Federal University of Uberlândia—UFU, Uberlândia 38.410-337, MG, Brazil

**Keywords:** bovine, oxidative stress, gene expression, methylation, cryopreservation

## Abstract

This study aimed to evaluate the effects of melatonin supplementation during bovine in vitro embryo production (IVEP) on embryonic development and quality, oxidative stress, lipid metabolism, mitochondrial activity, gene expression, DNA methylation patterns, and cryotolerance. Four treatments were tested: control (without melatonin), melatonin at maturation (IVM + Mlt), culture (IVC + Mlt), and both treatments (IMV/IVC + Mlt). Melatonin significantly improved blastocyst rate and developmental kinetics on D7, reduced ROS and intracellular lipid levels, and increased mitochondrial activity. The most significant effects were observed in the IVC + Mlt group. Melatonin modulated antioxidant (SOD1, Cat, and GSS) and epigenetic (TET1, TET3, and DNMT3A) genes, and although it did not alter lipid gene expression, it reduced lipid content. Methylation analysis showed hypomethylation patterns in repetitive regions (*Satellite I* and *LINE-1*), which were even more pronounced in the melatonin-treated groups. However, no significant differences were observed between treatments in terms of cryotolerance or apoptosis rates. These findings suggest that melatonin exerts positive multifactorial effects, regardless of the supplementation stage. In particular, its addition during the IVC phase appears to provide greater benefits to embryos by improving their quality.

## 1. Introduction

Several studies have shown that in vitro p0roduced embryos (IVEP) have lower developmental capacity than those generated in vivo. This reduction in quality is primarily associated with disturbances in cellular homeostasis, which are potentially caused by imbalances in key biological processes [1,2]. Among the several contributing factors, the culture environment plays a critical role in embryonic development. Parameters such as temperature, oxygen concentration, and culture medium composition must resemble physiological conditions to support optimal embryo development. Deviations from these conditions can negatively affect the viability and competence of embryos. Therefore, establishing in vitro maturation (IVM) and culture (IVC) systems that closely mimic the in vivo environment is essential to improve the efficiency and quality of IVEP embryos [3,4].

Considering that the generation of reactive oxygen species (ROS) is one of the most inevitable and impactful factors in in vitro systems, strategies to minimize oxidative stress are critical [5,6,7]. One such approach is supplementing the culture medium with antioxidants [8]. Antioxidants mitigate the harmful effects of ROS, which damage lipids, proteins, and nucleic acids. Excessive ROS levels are associated with increased apoptosis and impaired embryonic development [5]. Given the importance of antioxidant supplementation in IVEP media, several studies have explored various antioxidants, including quercetin [9], ascorbic acid [10], resveratrol [11], cysteamine [12], and glutathione [13], in maturation and/or culture media. Among these, melatonin has gained significant attention due to its antioxidant properties and broader physiological effects [14]. Melatonin (Mlt) (N-acetyl-5-methoxytryptamine) is a hormone synthesized by the pineal gland in mammals that not only regulates circadian rhythms but also plays important roles in immune modulation, metabolic regulation, and lipid metabolism [15,16].

The role of melatonin in combating oxidative stress is well documented, as it directly reduces ROS and indirectly increases glutathione production, thereby minimizing DNA damage and apoptosis [17,18,19,20]. In bovine IVEP systems, melatonin supplementation during IVM has been shown to improve oocyte maturation, cleavage, and blastocyst rates; increase glutathione levels; and improve mitochondrial function by reducing ROS accumulation [21,22,23]. Similar benefits have been observed in porcine models, where melatonin in IVM improves cumulus expansion, oocyte quality, and blastocyst yield and decreases ROS and apoptosis levels [11,18,19]. Similarly, in murine models, melatonin decreased ROS levels during IVM, resulting in improved embryonic quality and developmental outcomes [24,25]. Importantly, the beneficial effects of melatonin go beyond maturation, as its addition to the IVC medium has been associated with improved cleavage, blastocyst, and hatching rates; elevated glutathione levels; and improved mitochondrial distribution in several species [16,20,26], which supports the potential of melatonin to positively influence various stages of embryonic development.

In addition to its well-established antioxidant properties, melatonin is associated with lipid metabolism modulation and epigenetic reprogramming, particularly in murine and porcine models. In pigs, melatonin supplementation during IVM reduces lipid droplet size, increases the transcription of genes related to lipogenesis and lipolysis, and increases fatty acid content, mitochondrial activity, and ATP levels [27,28,29], reinforcing its multifaceted role. Similarly, studies on porcine embryos produced by somatic cell nuclear transfer (SCNT) have shown that melatonin can improve developmental outcomes by epigenetic reprogramming, such as DNA methylation and histone acetylation, as well as by reducing oxidative stress and apoptosis [30,31]. However, to our knowledge, the effects of melatonin on lipid metabolism have been scarcely investigated, and studies on its influence on epigenetic mechanisms have been limited to histone acetylation analysis of SCNT embryos [32].

Although the antioxidant effects of melatonin and its overall benefits in bovine IVEP are widely recognized, its role in lipid metabolism and epigenetic reprogramming in this species remains poorly understood. Furthermore, although positive effects have been demonstrated when melatonin is added separately during IVM or IVC, the impact of continuous supplementation throughout the IVEP process is yet to be fully elucidated. Further investigations are needed to clarify the mechanisms of action of melatonin and to establish the most effective supplementation strategy for improving IVEP outcomes in cattle.

Based on the combined effects of melatonin, we hypothesized that continuous supplementation during IVEP might improve bovine embryo yield and quality more effectively than stage-specific supplementation. To test this hypothesis, we evaluated the effects of continuous melatonin supplementation during bovine IVEP on embryonic development, kinetics, oxidative stress, lipid content, cryotolerance, gene expression, and DNA methylation.

## 2. Materials and Methods

All reagents used in this study were obtained from Sigma-Aldrich (St. Louis, MO, USA) unless otherwise specified.

All biological materials used in the present study were obtained from ovaries collected from slaughterhouses, and all procedures were performed in accordance with the Brazilian Animal Protection Law (No. 10, 468 (2020)).

This study aimed to evaluate whether supplementation of in vitro maturation (IVM) and/or culture (IVC) media with melatonin improves bovine embryonic development and quality. Oocytes were subjected to four experimental conditions: (1) (CT), in which both maturation and culture were conducted in standard media without melatonin; (2) IVM + Mlt, in which only the maturation medium was supplemented with melatonin; (3) IVC + Mlt, in which only the culture medium was supplemented; and (4) IVM/IVC + Mlt, in which both the maturation and culture media were supplemented with melatonin. In all treatments, melatonin was added at a concentration of 10^−9^ M [22].

### 2.1. Immature Oocyte Collection

Ovaries were collected from local slaughterhouses immediately after slaughter and transported to the laboratory in 0.9% saline solution (NaCl 0.9%) with streptomycin sulfate (100 μg/mL) and penicillin G (100 IU/mL), at 32–36 °C.

Cumulus-oocyte complexes (COCs) were aspirated from follicles with diameters ranging from 3 to 8 mm using an 18 G hypodermic needle attached to a 10 mL syringe. After aspiration, the pellet containing the oocytes was allowed to settle at the bottom of a conical tube for at least 10 min. Subsequently, 10 mL of the follicular fluid supernatant was centrifuged at 700× *g* for 5 min for COC retrieval and selection. Only COCs with three or more layers of cumulus cells and a uniform cytoplasm, or those exhibiting fine granulation, were selected for the experiments.

### 2.2. In Vitro Embryo Production

The in vitro production of bovine embryos was performed as previously described. After COC selection, 25–30 oocytes were transferred to drops of maturation medium, covered with mineral oil, and cultured for 22–24 h. The in vitro maturation medium (IVM) consisted of TCM-199 medium with Earl salts (Gibco^®^ BRL, Burlington, ON, Canada), supplemented with 10% fetal bovine serum (FBS), 0.01 UI/mL of follicle stimulating hormone (FSH), 0.1 mg/mL of L-glutamine, 0.075 mg/mL of amikacin, and 0.1 μM of cysteamine.

After maturation, the COCs were transferred to drops of fertilization medium (TALP) covered with mineral oil [33]. For in vitro fertilization (IVF), semen from a previously tested Nellore bull (Bos taurus indicus) was processed using a discontinuous Percoll gradient (GE^®^ Healthcare, PisCataway, NJ, USA) [33]. Selected spermatozoa were added to the fertilization drop at a final concentration of 1 × 10^6^ spermatozoa/mL and co-incubated with mature oocytes for 18–20 h. The IVF Day was considered day zero (D0). After fertilization, presumptive zygotes were partially denuded and transferred to drops of culture medium (SOF) [34], supplemented with essential and non-essential amino acids, 0.35 mM sodium citrate, 2.8 mM myo-inositol, and 4 mg/mL Bovine Serum Albumin (BSA), then covered with mineral oil. Throughout the process, the culture was performed at 38.5 °C in an atmosphere with 5% CO_2_ in the air and controlled humidity.

Embryo development was assessed on Day 2 (D2) for cleavage and on Days 6 and 7 (D6 and D7) for blastocyst formation and developmental kinetics. On D7, the expanded blastocysts were used for further analyses, namely, gene expression, DNA methylation profiling, staining with H_2_DCFDA, BODIPY 493/503, MitoTracker Deep Red, and Hoechst 33342, and cryotolerance assessment.

### 2.3. Detection of ROS

Intracellular ROS levels in expanded blastocysts were quantified using 6-carboxy-2′,7′-dichlorodihydrofluorescein diacetate (H_2_DCFDA) (Thermo Fisher, Waltham, MA, USA) [29]. H_2_DCFDA was dissolved in dimethyl sulfoxide (DMSO) and stored as a stock solution. For use, H_2_DCFDA was diluted in Phosphate-Buffered Saline (PBS) with 1% BSA to a final concentration of 50 µM. The BX were washed three times in PBS-BSA and incubated in a 400 μL drop of H_2_DCFDA for 30 min at 38.5 °C in the dark. Subsequent evaluations were performed under a fluorescence microscope (Zeiss AxioPlot, Carl Zeiss Microscopy LLC, White Plains, NY, USA). To obtain and quantify the fluorescence signal intensities (pixels), a wavelength of 488 nm was used, with an excitation power of 9.14%, and emissions were recorded in the range of 475–535 nm. Fluorescence intensity of ROS was quantified in ImageJ^®^ software (National Institutes of Health, Bethesda, MD, USA) (version 1.8.0_345) by measuring the mean pixel intensity within each expanded blastocyst. All images were acquired under identical microscope settings, and results are presented as mean pixel intensity per expanded blastocyst ± SD.

### 2.4. Evaluation of Mitochondrial Activity, Lipid Droplet Accumulation, and Total Cell Number

The expanded blastocysts were washed three times in PBS supplemented with 0.3% polyvinylpyrrolidone (PVP) and incubated for 30 min with Mito Tracker Deep Red FM at 400 nM (diluted in SOF) at a temperature of 35 to 37 °C. Subsequently, the BX were washed three times in PBS-PVP, fixed for 1 h in 4% paraformaldehyde, washed three times in PBS-PVP, and stored at 4 °C in 1% paraformaldehyde for 7 days. The embryos were then washed three times in PBS-PVP and incubated for 30 min in PBS supplemented with 0.2% Triton at room temperature, washed three times in PBS-PVP, and then stained with BODIPY 493/503 (Molecular Probes, Eugene, OR, USA) at a concentration of 20 μg/mL. This was diluted in 50 μL of absolute ethanol and then further diluted in 950 μL of PBS for one hour. The embryos were washed three times in PBS-PVP and placed in 35 mm plates in drops of 8 μL of PBS-PVP and then visualized under a Leica Sp8 confocal microscope (New Orleans, LA, USA).

All samples were analyzed and photographed at 20 X using an argon laser at 488 nm and a fluorescence spectrum between 495 and 505 nm to visualize the lipid droplets. For mitochondrial activity evaluation, a 638 nm laser was used with emission/excitation at 644/665 nm. The embryos were subjected to up to 50 cross-sectional slices spaced 4 mm apart. Z-stacking was used to create images of overlapping sections. The average number of pixels was calculated to determine the mitochondrial activity. For lipid droplet evaluation, after creating the final image of each embryo, the image was adjusted to grayscale (8-bit image), and the lipid area (number of pixels) was measured relative to the total area of the oocyte (μm^2^). Both evaluations were performed using the ImageJ software (National Institutes of Health).

After evaluation with Mito Tracker and BODIPY, the embryos were washed three times in PBS-PVP and stained with Hoechst 33342 (10 mg/mL) for 15 min in the dark. Finally, they were washed three times in PBS-PVP and mounted on slides in drops of 8 μL of PBS-PVP. The total number of cells was counted under a fluorescence microscope (Zeiss^®^ Axioplot).

Mitochondrial activity was evaluated by measuring the mean fluorescence intensity (in pixels) per blastocyst using ImageJ software. All images were acquired using identical microscope settings, and each blastocyst was manually selected as the region of interest (ROI) to calculate the mean pixel intensity. Lipid droplet accumulation was quantified as the percentage of lipid-stained area relative to the total area of the blastocyst. This analysis was performed using ImageJ through grayscale image conversion and threshold adjustment to isolate the fluorescent lipid regions.

### 2.5. RT-qPCR

For the quantification of relative mRNA abundance, four pools of 25 D7 BX from each treatment were used. The embryos were then placed in microtubes with 5 μL of PBS and stored at −80 °C until RNA extraction. Total RNA was extracted from blastocysts using an RNeasy Plus Micro Kit (Qiagen, Hilden, Germany) according to the manufacturer’s instructions. The total RNA was then used for cDNA synthesis with the GoScript Reverse Transcriptase Kit (Promega^®^, Madison, WI, USA), with Oligo-dT (0.5 μg/μL) and random primers (0.5 μg/μL) in a final volume of 30 μL, following the manufacturer’s instructions. Reactions were carried out at 70 °C for 5 min, followed by a 5 min annealing step at 25 °C, a 60 min extension at 42 °C, and enzyme inactivation at 70 °C for 15 min. qPCR was performed using the 7500 Fast Real-Time PCR System (Applied Biosystems, Foster City, CA, USA). qPCR was performed using the GoTaq qPCR Master Mix Kit (Promega). The efficiencies were calculated using serial dilutions (1/4 dilutions). Each sample was analyzed in triplicate, and the specificity of each amplicon was determined based on melting curve analysis and amplicon size on an agarose gel. Reactions were performed in a final volume of 25 μL using cDNA equivalent to 0.59 D7 BX/reaction. The reactions were optimized to obtain maximum amplification efficiency for each gene (Table 1). Amplification conditions were 95 °C for 1 min, followed by 50 cycles of denaturation at 95 °C for 15 s and annealing and extension at 60 °C for 1 min, with a dissociation (melting curve) at 60–95 °C.

The nomenclature, primer sequences and concentrations, amplicon sizes, GenBank accession numbers, primer efficiencies, and melting temperatures are listed in Table 1. All genes were analyzed equally, following the above protocol, for genes related to epigenetic reprogramming (DNMT3A, DNMT3B, TET1, TET2, and TET3), oxidative stress-related enzyme genes (SOD1, GSS, and CAT), and lipid metabolism (CPT1A, PLIN2, and PPARγ). GAPDH was chosen as the reference gene for data normalization [35]. The relative expression of each gene was calculated by the ΔΔCt method with efficiency correction by the Pfaffl method [36].

### 2.6. DNA Methylation Analysis

DNA methylation analysis of *Satellite I* of the bovine testicle Bos taurus (*Satellite I*) and the retrotransposon long interspersed element-1 (*LINE-1*) was performed using bisulfite PCR. These regions were selected because they represent a large proportion of the eukaryotic genome, as they are highly repetitive sequences that are widely distributed throughout the genome. Thus, methylation of these regions may reflect the global epigenetic patterns of genomic DNA [37].

Three pools of five D7 BX produced by the control, IVM + Mlt, IVC + Mlt, and IVM/IVC + Mlt treatments were used for DNA methylation analysis. First, the embryos were removed from the culture medium and washed three times with calcium- and magnesium-free PBS. The embryos were then placed in microtubes with 5 μL of PBS and stored at −80 °C until genomic DNA extraction. Genomic DNA was isolated from blastocysts as previously reported by Mendonça et al., 2015 [38]. DNA samples were stored at −20 °C until further use. Primers were designed to flank the CpG islands in repetitive DNA sequences from *Satellite I* and *LINE-1*. The primer sequences, GenBank accession numbers, CpG numbers, and amplicon sizes are listed in Table 2.

DNA samples were treated with sodium bisulfite using the EZ DNA Methylation- Lightning™ Kit (Zymo Research, Orange, CA, USA) according to the manufacturer’s protocol. Sodium bisulfite-treated DNA samples were subjected to PCR amplification in duplicate. PCR of these regions was performed in a total volume of 20 μL comprising 1X Taq buffer, 1.5 and 1.0 mM MgCl_2_ (*Satellite I* and *LINE-1*, respectively), 0.4 mM dNTPs, 1U of Platinum™ Taq DNA Polymerase (Invitrogen™, Carlsbad, CA, USA), 0.25 µM of each primer (forward and reverse), and 2 µL of bisulfite-treated DNA. Amplification was carried out with an initial denaturation step at 94 °C for 3 min, followed by 45 cycles of denaturation at 94 °C for 45 s, annealing at 45 °C and 62 °C (for *Satellite I* and *LINE-1*, respectively) for 40 s, and extension at 72 °C for 1 min, followed by a final extension at 72 °C for 20 min.

After PCR, amplicons were purified from agarose gels using the Wizard™ SV Gel and PCR Clean-Up System kit (Promega™, Madison, WI, USA) according to the manufacturer’s instructions. Purified amplicons were cloned into the TOPO TA Cloning vector (PCR 2.1-TOPO^®^ vector system, Invitrogen, Carlsbad, CA, USA) and transferred into DH5α cells using a heat shock protocol. Plasmid DNA was isolated using the PureYield™ Plasmid Miniprep System kit (Promega™, Madison, WI, USA), and individual clones were sequenced using BigDye^®^ (Thermo Fisher Scientific, Waltham, MA, USA) cycle sequencing chemistry and an ABI3100 automated sequencer.

Electropherogram quality was analyzed using electropherogram quality analysis (http://lbi.cenargen.embrapa.br/phph/ accessed on 10 April 2025), and methylation patterns were processed using the Quantification Tool for Methylation Analysis (QUMA, http://quma.cdb.riken.jp/top/index.html accessed on 20 April 2025) [39]. DNA sequences were compared with GenBank reference sequences (accession numbers are shown in Table 2). Only sequences that originated from clones with ≥90% identity and cytosine conversion were used (n = 348: *LINE-1*: 188; *Satellite I*: 160). The conversion of non-CpG cytosines was used to calculate the efficiency of the bisulfite treatment, and the methylation pattern of CpG cytosines was used to identify individual clones from different DNA templates. Thus, each DNA clone was determined by its methylation pattern and considered a replicate. A comparative methylation analysis of each CpG site was performed between the treatment groups. An additional analysis was also conducted to compare the number of hypomethylated alleles (≤20%) and hypermethylated alleles (≥80%) among the treatments. Because methylation of specific CpG sites may interfere with transcription factor binding and subsequently affect gene expression, the TFBIND INPUT software (https://tfbind.hgc.jp/ accessed on 12 May 2025) was used to identify potential transcription factor-binding sites within these regions.

### 2.7. Vitrification and Warming of Expanded Blastocysts

To elucidate the viability and embryonic quality regarding the cryotolerance of the embryos, vitrification was performed using Cryotop^®^, as previously described [40], with some modifications, in about 60 BX on D7. The vitrification and handling solutions were preheated to 38 °C prior to use. A maintenance solution (SM) consisting of TCM-199 with Hank’s salts, L-glutamine, and HEPES 25 mM (Gibco BRL, Burlington, ON, Canada) supplemented with 20% fetal bovine serum (FBS) was used to manipulate the embryos during vitrification and served as the basis for the vitrification solutions.

Embryos from each treatment group were exposed for 9 min to vitrification solution 1 (VS1), also known as the equilibration solution, containing 7.5% ethylene glycol (EG) and 7.5% dimethyl sulfoxide (DMSO) dissolved in SM for 9 min. Subsequently, they were transferred to vitrification solution 2 (VS2), or the final vitrification solution, composed of 15% EG, 15% DMSO, and 0.5 M sucrose diluted in SM, for 45 s.

A group of up to 5 embryos was positioned on a vitrification device (WTA—Soluções para Reprodução Animal, Cravinhos, SP, Brazil), and all excess vitrification solution was removed until only a thin layer of less than 0.1 μL remained. Finally, the device containing the embryos was immediately immersed in liquid nitrogen (N_2_).

The process of warming and rehydration of the embryos was carried out at a controlled temperature of 38.5 °C. and immediately immersed for 1 min in the first thawing solution (DV1) containing 1 M sucrose diluted in SM. They were then transferred to the second thawing solution (DV2), containing 0.5 M sucrose diluted in SM, where they remained for 3 min. The embryos were then transferred to SM and kept for a minimum of 5 min and a maximum of 10 min until they were transferred back to the culture drop corresponding to the experimental group and were cultured for another 24 h. Reexpansion, hatching, and degeneration were evaluated at 12 and 24 h post-thawing. On each warming and evaluation day, a fresh control group not subjected to the vitrification process and at the same developmental stage as the vitrified embryos was included in all assessments.

### 2.8. Total Cell Number and Percentage of Apoptotic Cells

After 24 h of thawing, reexpanded blastocysts were analyzed for total and apoptotic cell numbers according to Fidelis et al., 2020 [41]. Fresh embryos were used as controls for vitrified embryos. The Click-iT^®^ TUNEL Alexa Fluor^®^ kit (Thermofisher, Waltham, MA, USA) was used for labeling, with positive control (DNase), negative, and experimental groups submitted to the TdT reaction and later to the Click-iT reaction. Subsequently, the embryos were stained with Hoechst 33342 and analyzed using a fluorescence microscope. Filters were used to capture excitation wavelengths of 495/519 nm for Alexa Fluor 488 and 350/461 nm for Hoechst 33342. For each blastocyst, the total number of cells (Hoechst staining 33342) and total number of apoptotic cells (TUNEL) were determined. The percentage of apoptotic cells was calculated from these values.

### 2.9. Statistical Analyses

Analyses were performed using GraphPad Prism 9 (GraphPad Software, San Diego, CA, USA) and the QUMA software (2008-2019). Data on cleavage rates, blastocyst development on D6 and D7, the kinetics of embryonic development, reexpansion, hatching, and degeneration were compared between the experimental groups using the chi-square test. ROS levels, mitochondrial activity, lipid droplet area, total number of cells, total number of apoptotic cells, and percentage of apoptosis were compared between treatments using one-way analysis of variance (ANOVA), followed by Tukey’s test.

Gene expression data were compared between the experimental groups by means of analysis of variance (ANOVA) followed by the Tukey–Kramer multiple comparison test or, when comparing two by two, by the *t*-test of independent samples. The results are presented as mean ± standard deviation (SD). Global methylation of each region was compared using the Mann–Whitney test. In addition, a comparative methylation analysis of each CpG site was performed using Fisher’s exact test. In the hyper/hypomethylation analysis of the alleles, the groups were classified as high (≥80%) and low (≤20%) methylation; the data from these analyses were compared between the experimental groups using the chi-square test or Fisher’s exact test when compared two by two.

## 3. Results

### 3.1. Effect of Melatonin on Embryo Production and Development

The effects of melatonin on embryonic production and developmental kinetics were evaluated using 5806 oocytes, from which 2373 embryos were produced, resulting in an average production rate of 40.87% in twenty-one replicates.

As shown in Table 3, no differences were observed between the treatments on D2 and D6. However, on D7, the control group exhibited a lower rate of embryo production than melatonin-treated groups. Among the melatonin treatments, the IMV group was similar (*p* > 0.05) to the IVC and IVM/IVC groups, whereas the IVC and IVM/IVC groups differed from each other (*p* < 0.05).

Regarding the kinetics of embryonic development shown in Table 4, differences (*p* < 0.05) were observed in the early blastocyst (EB) and blastocyst (BL) stages on Day 6 (D6) between the control group and all groups treated with melatonin. On D6, at the BX stage, the IVC + Mlt group was the only melatonin treatment that differed (*p* < 0.05) from the control. However, by D7, embryos from the melatonin-treated groups exhibited a faster development, as evidenced by a higher percentage (*p* < 0.05) of hatched blastocysts compared to the control group.

### 3.2. Effect of Melatonin on ROS Levels

**Figure 1:**
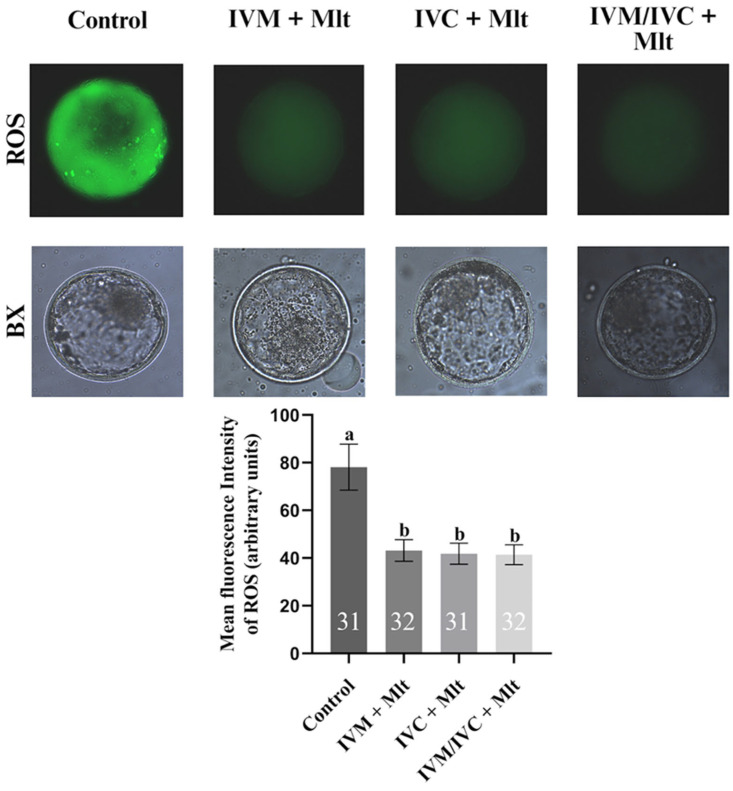
Intracellular reactive oxygen species (ROS) levels assessed by H_2_DCFDA in different treatment groups: (1) Control: no melatonin added; (2) IVM + Mlt: melatonin added during in vitro maturation; (3) IVC + Mlt: melatonin added during in vitro culture; (4) IVM/IVC + Mlt: melatonin added during both in vitro maturation and culture. ^a,b^ Values with different superscript letters between columns are significantly different (*p* < 0.05), as determined by ANOVA. The numbers above the bars indicate the number of stained embryos assessed in each evaluation.

As shown in Figure 1, ROS levels, measured by the fluorescence intensity of the H_2_DCFDA dye, were lower (*p* < 0.05) in the melatonin-supplemented groups: IVM + Mlt (43.2 ± 4.5%), IVC + Mlt (41.8 ± 4.4%), and IVM/IVC + Mlt (41.4 ± 4.1%) compared to the group (78.1 ± 9.7%).

### 3.3. Effect of Melatonin on Mitochondrial Activity, Lipid Droplet Accumulation, and Total Cell Number

**Figure 2:**
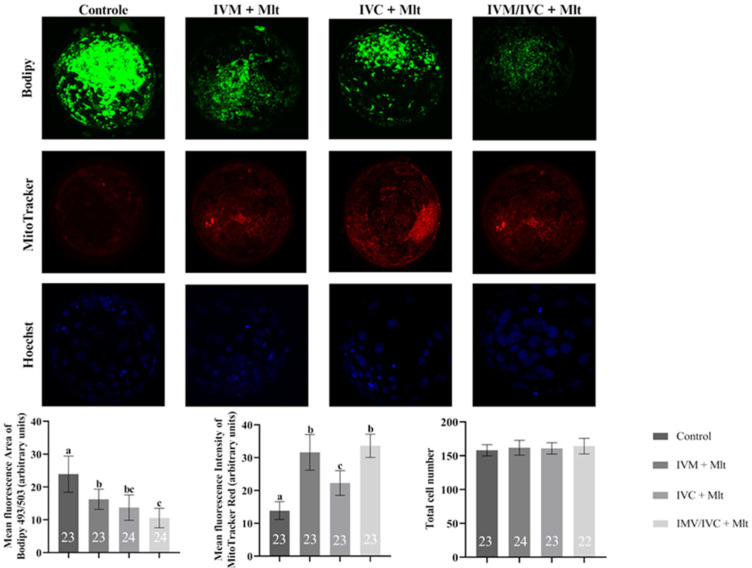
Intracellular lipid droplet levels in blastocysts assessed by BODIPY 493/503, mitochondrial activity levels evaluated by Mito Tracker Deep Red, and total cell count by Hoechst 33342 in different groups: (1) Control: no melatonin added; (2) IVM + Mlt: melatonin added during in vitro maturation; (3) IVC + Mlt: melatonin added during in vitro culture; (4) IVM/IVC + Mlt: melatonin added during both in vitro maturation and culture. ^a,b,c^ Values with different superscript letters between columns are significantly different (*p* < 0.05), as determined by ANOVA. The numbers above the bars indicate the number of stained embryos assessed in each evaluation.

The mitochondrial activity, lipid droplet accumulation, and total cell count results are presented in Figure 2. Lipid content was lower in all melatonin-treated groups than in the group (23.91 ± 6.37%). The greatest reduction was observed in the group in which melatonin was present during the two stages, IVM and IVC (10.56 ± 3.02%). However, the group in which melatonin was supplemented only during IVC (13.72 ± 4.56%) showed an intermediate reduction, which was statistically similar to the other two melatonin-treated groups, in IVM alone (16.26 ± 3.36%) and present in both stages.

Unlike the lipid content, mitochondrial activity was higher in all melatonin-treated groups than in the Control. The highest mitochondrial activity was observed in the groups in which melatonin was present during IVM (IVM + Mlt and IVM/IVC + Mlt), which were statistically similar (*p* > 0.05). However, both groups showed significantly higher activity (*p* < 0.05) than the IVC + Mlt group, with an intermediate increase.

Regarding the total cell count, which was assessed using Hoechst 33342, no significant differences (*p* > 0.05) were observed among the Control, IVM + Mlt, IVC + Mlt, and IVM/IVC + Mlt groups.

### 3.4. Effect of Melatonin on Embryo Cryotolerance

Regarding embryo cryotolerance, no differences (*p* > 0.05) were observed between the treatment groups (*p* > 0.05) in terms of embryonic development, degeneration, and re-expansion at 12 or 24 h after thawing (Table 5).

Similarly, no differences (*p* > 0.05) were detected among treatments in the total cell number and percentage of apoptotic cells (Table 6). All these analyses were also performed in the fresh embryo group (control of vitrified embryos) and were included in the statistical analyses (Table 5 and Table 6).

### 3.5. Quantification of Relative mRNA Abundance

**Figure 3:**
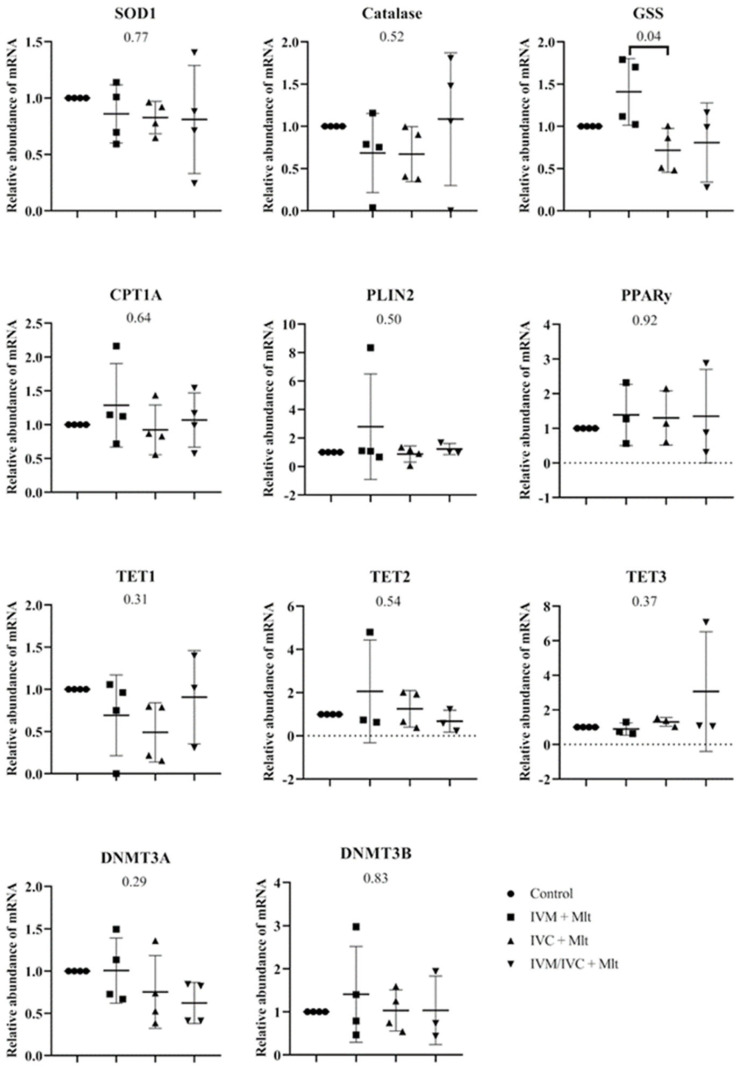
Transcript levels of genes related to epigenetic reprogramming—*DNMT3A* (DNA methyltransferase 3 alpha), *DNMT3B* (DNA methyltransferase 3 beta), *TET1* (Ten-Eleven Translocation methylcytosine dioxygenase 1), *TET2* (Ten-Eleven Translocation methylcytosine dioxygenase 2), and *TET3* (Ten-Eleven Translocation methylcytosine dioxygenase 3); oxidative stress—*SOD1* (Superoxide Dismutase 1), *GSS* (Glutathione Synthetase), and *CAT* (Catalase); and lipid metabolism—*CPT1A* (Carnitine Palmitoyltransferase 1A), *PLIN2* (Perilipin 2), and *PPARγ* (Peroxisome Proliferator-Activated Receptor Gamma), were evaluated in bovine day-7 expanded blastocysts (D7 BX). For each treatment, four pools of 25 D7 BX were used and analyzed in triplicate. The groups were compared by ANOVA.

Figure 3 shows the mRNA levels of *SOD1*, *GSS*, *CAT*, *CPT1A*, *PLIN2*, *PPARγ*, *TET1*, *TET2*, *TET3*, *DNMT3A*, and *DNMT3B* in blastocysts. Among all the genes evaluated, only *GSS* showed a significant difference (*p* < 0.05) between the IVM + Mlt and IVC + Mlt groups, with higher transcript levels in the IVM + Mlt group.

**Figure 4:**
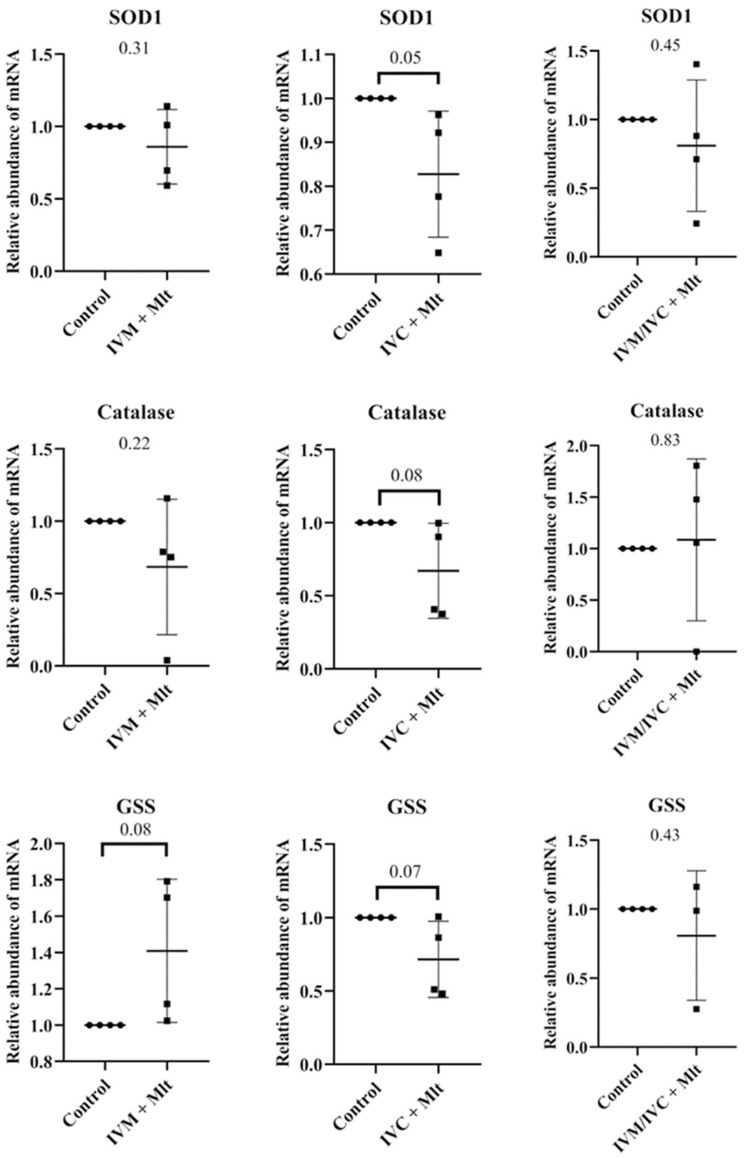
Transcript levels of genes related to oxidative stress, *SOD1* (Superoxide Dismutase 1), *GSS* (Glutathione Synthetase), and *CAT* (Catalase) were evaluated in bovine day-7 expanded blastocysts (D7 BX). For each treatment, four pools of 25 D7 BX were used and analyzed in triplicate. The melatonin-containing groups were compared with the control using Student’s *t*-test.

Comparison of each melatonin-treated group individually to the control revealed distinct expression patterns of oxidative stress-related genes (*SOD1*, *CAT*, and *GSS*) (Figure 4). The IVC + Mlt group showed lower transcript levels of *SOD1*, *CAT*, and *GSS* than the control (*p* < 0.10), whereas the IVM + Mlt group exhibited increased expression of *GSS* (*p* < 0.10).

**Figure 5:**
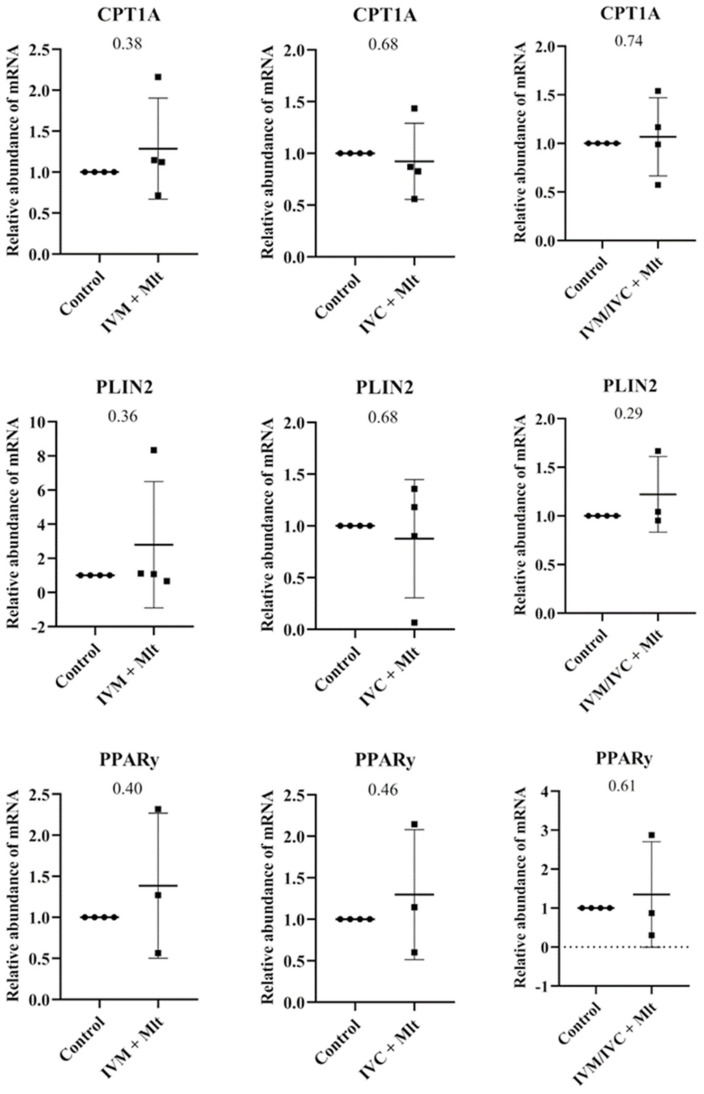
Transcript levels of genes related to lipid metabolism, *CPT1A* (Carnitine Palmitoyltranferase 1A), *PLIN2* (Perilipin 2), and *PPAR*γ (Peroxisome Proliferator-Activated Receptor Gamma), were evaluated in bovine day-7 expanded blastocysts (D7 BX). For each treatment, four pools of 25 D7 BX were used and analyzed in triplicate. The melatonin-containing groups were compared with the control using Student’s *t*-test.

The genes related to lipid metabolism (Figure 5), Carnitine palmitoyltransferase 1A (*CPT1A*), Perilipin 2 (*PLIN2*), and Gamma Peroxisome Proliferator-Activated Receptor (*PPARγ*) showed no statistically significant differences between the control group and each of the melatonin-treated groups (*p* > 0.05).

**Figure 6:**
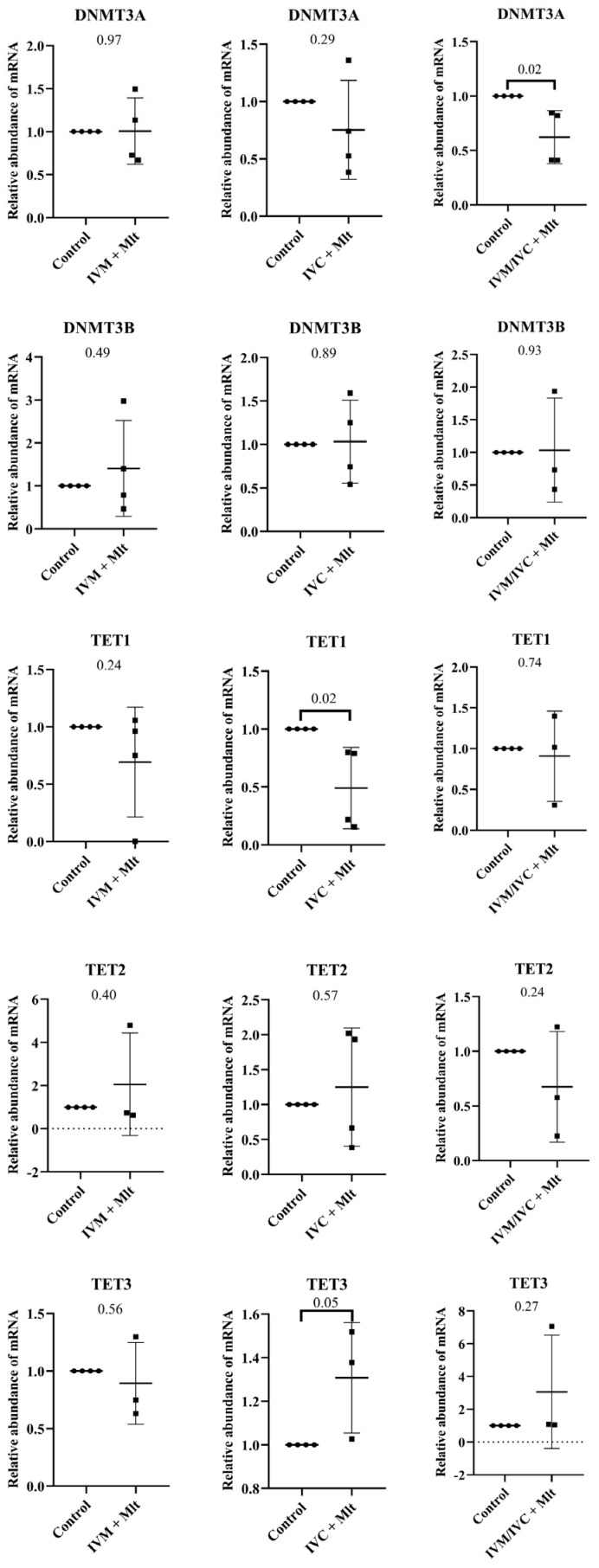
Transcript levels of genes related to epigenetic reprogramming, *DNMT3A* (DNA methyltransferase 3 alpha), *DNMT3B* (DNA methyltransferase 3 beta), *TET1* (Ten-Eleven Translocation methylcytosine dioxygenase 1), *TET2* (Ten-Eleven Translocation methylcytosine dioxygenase 2), and *TET3* (Ten-Eleven Translocation methylcytosine dioxygenase 3), were evaluated in bovine day-7 expanded blastocysts (D7 BX). For each treatment, four pools of 25 D7 BX were used and analyzed in triplicate. The melatonin-containing groups were compared with the control using Student’s *t*-test.

In contrast, the genes related to epigenetic reprogramming (TET1, TET2, TET3, DNMT3A, and DNMT3B) (Figure 6) showed differences between the control group and each of the melatonin-treated groups. The mRNA levels of *TET1* were lower in the IVC + Mlt group (*p* < 0.05) compared to the control group, whereas *TET3* levels were higher in the IVC + Mlt group (*p* ≤ 0.05). Additionally, *DNMT3A* mRNA levels were lower in the IVM/IVC + Mlt group than in the control group (*p* < 0.05).

### 3.6. Effect of Melatonin on DNA Methylation Profiles

**Figure 7:**
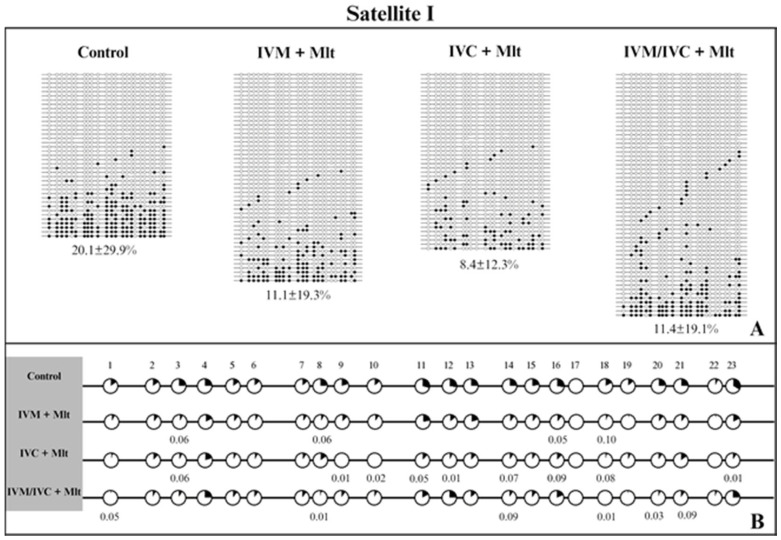
DNA methylation profile of the *Satellite I* gene. (**A**) Global methylation profile of the *Satellite I* region between the control and melatonin groups. (**B**) Comparative analysis of methylation by CpG sites between the control and melatonin groups. Each row represents an individual DNA clone, and each circle represents a CpG dinucleotide. The black circles represent methylated cytokines, and the white circles represent unmethylated cytosines. The percentage of DNA methylation for each treatment (control; IVM + Mlt; IVC + Mlt; IMV/IVC + Mlt) is represented as the mean ± standard deviation of the mean. (1–23) represents the number of CpGs. Values of *p* ≤ 0.10 are shown. The Mann–Whitney test was used for global methylation analysis, while Fisher’s exact test was used for CpG site methylation analysis.

**Figure 8:**
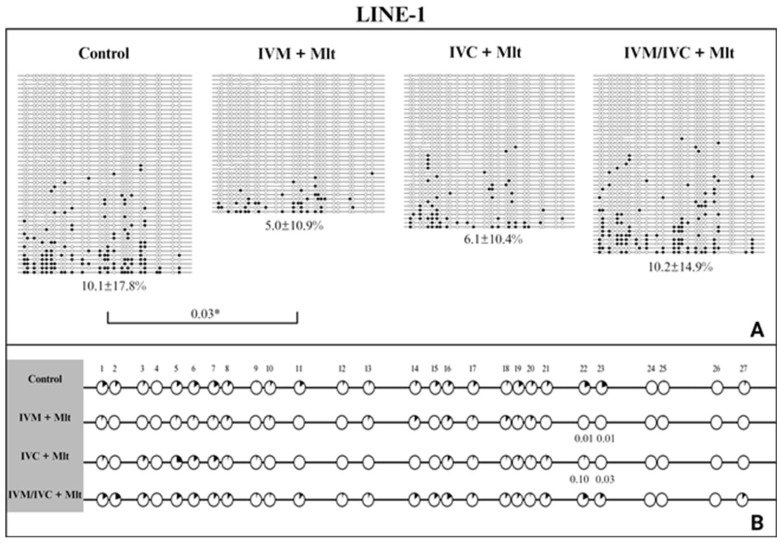
DNA methylation profile of the *LINE-1* gene. (**A**) Global methylation profile of the *Satellite I* region between the control and melatonin groups. Values of *p* ≤ 0.05 are shown as *. (**B**) Comparative analysis of methylation by CpG sites between the control and melatonin groups. Each row represents an individual DNA clone, and each circle represents a CpG dinucleotide. The black circles represent methylated cytokines, and the white circles represent unmethylated cytosines. The percentage of DNA methylation for each treatment (IVM + Mlt; IVC + Mlt; IMV/IVC + Mlt) is represented as mean ± standard deviation of the mean. (1–27) represents the number of CpGs. Values of *p* ≤ 0.10 are shown. The Mann–Whitney test was used for global methylation analysis, while Fisher’s exact test was used for CpG site methylation analysis.

**Figure 9:**
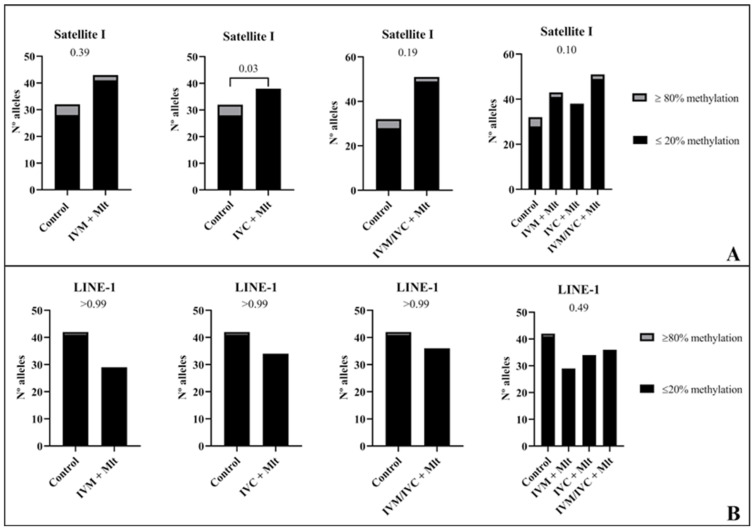
Comparison between frequency/number of hyper/hypomethylated alleles (80/20%). (**A**): In the *Satellite I* region, alleles with methylated CpG ≥ 18 were classified as ≥ 80% and alleles with methylated CpG ≤ 5 were classified as ≤ 20%. (**B**): In the *LINE-1* region, in turn, alleles with methylated CpG ≥ 22 were classified as ≥ 80% and alleles with methylated CpG ≤ 5 were classified as ≤ 20%. The data from these analyses were compared among the experimental groups using the chi-square test or Fisher’s exact test when comparing the control group with groups with melatonin.

We evaluated the methylation profiles of two repetitive DNA sequences (*Satellite I* and *LINE-1*). The results are shown in Figure 7, Figure 8 and Figure 9. The global methylation pattern of the SAT-I gene (Figure 7A) was not different between the melatonin-treated and control groups (*p* > 0.05); all groups exhibited a hypomethylation pattern. Similarly, for the *LINE-1* gene (Figure 8A) exhibited a hypomethylation pattern across all treatments. However, compared to the control (10.1% ± 17.8), the IVM + Mlt group exhibited significantly less methylation (*p* < 0.05) (Figure 8A).

Analyzed the percentage of methylation at each CpG site individually for *Satellite I* (Figure 7B). In all situations where differences occurred, the melatonin-treated groups exhibited less methylated CpG than the control group (*p* < 0.10) (Figure 7B). In *LINE-1* cells (Figure 8B), the group exposed to melatonin during both maturation and culture (IVM + Mlt) differed from the control at CpG sites 22 and 23, with the melatonin-treated group showing significantly lower DNA methylation (*p* < 0.05).

Moreover, in the comparative analysis of methylation by CpG of *Satellite I* and *LINE-1* (Figure 7B and Figure 8B, respectively), CpG 18 and CpGs 22/23 of *Satellite I* and *LINE-1*, respectively, were identified as differentially methylated cytosines (DMCs) between the control and treatment IVC + Mlt groups. These specific CpGs may influence the binding of transcription factors and, consequently, affect gene expression. The TFBIND INPUT software (https://tfbind.hgc.jp/ accessed on 12 May 2025) was used to search for transcription factors in these regions. In all three of these DNA domains that contain these CpGs, the consensus sequence was M00211 V$PADS (NGTGGTCTCGAGGCCACG), showing 0.87 similarity (on a scale of 0.0–1.0) of the studied sequence with the transcription factor binding site. This sequence corresponds to the DNA-binding domain of the PAX protein (paired domain transcription factors) according to the TRANSFAC^®^ database.

The DMC of *Satellite I* (CpG 18) showed less DNA methylation in all three melatonin-containing treatments than in the control (*p* < 0.10) (Figure 7B). The DMCs of *LINE-1* (CpG 22 and 23) also showed lower DNA methylation than the control, but only in the IVM + Mlt and IVC + Mlt groups (Figure 8B).

In the analysis of hyper/hypomethylated alleles (80/20) (Figure 9), *Satellite I* (Figure 9A) showed that the IVC + Mlt treatment had a higher number of hypomethylated alleles with methylation ≤ 20% when compared to the control (*p* < 0.05). In the *LINE-1* (Figure 9B), the alleles in all the treatments containing melatonin and in the control were hypomethylated (≤20%).

## 4. Discussion

Melatonin has emerged as a promising option to improve IVEP efficiency because of not only its known antioxidant potential but also its additional beneficial effects on metabolism and epigenetic mechanisms. Therefore, in this study, we aimed to examine these effects in bovine embryos and determine the stage during IVEP when melatonin offers the most optimal benefits.

Initially, we evaluated blastocyst production following the different treatments. Melatonin supplementation did not affect the cleavage rate or blastocyst formation on D6 but had a significant effect on D7, where all melatonin-treated groups exhibited a higher blastocyst rate than the control. This may be due to the time-dependent nature of melatonin’s actions, which require more time to manifest. In addition, the use of BSA in the culture medium, rather than fetal bovine serum, may have slowed the overall development of the blastocyst, causing the differences to become evident only at later stages. This finding is in line with previous studies, which have shown improvements with melatonin use during bovine IVM [23,26,42,43] and IVC [20,44]. Although few studies have assessed the effects of melatonin supplementation throughout the IVEP process, our findings demonstrate that its positive effect on blastocyst production is maintained regardless of the timing or duration of supplementation.

These beneficial effects of melatonin can be primarily attributed to its antioxidant properties, as it reduces ROS by directly neutralizing free radicals, hydroxyl (^•^OH), superoxide (O_2_•^−^), and hydrogen peroxide (H_2_O_2_), which are the most abundant and harmful to the biological environment. During IVC, particularly in the embryonic genome activation (EGA) phase, melatonin mitigates oxidative damage, regulates the cell cycle, and controls apoptosis, thereby improving embryo viability and quality [5,6,7]. However, although the IVM + Mlt and IVM/IVC + Mlt groups performed better than the control group, they were inferior to the IVC + Mlt group in terms of embryo production, suggesting that melatonin exposure for only 24 h, as occurs in IVM, may be insufficient to achieve the same benefits observed with supplementation during IVC, and that continued exposure to melatonin during IVM and IVC offers no additional advantages, contrary to our initial expectations. Instead, prolonged exposure may have induced a negative effect, possibly due to excessive modulation of metabolic pathways and cell signaling, a phenomenon previously observed in a study that used melatonin in IVM and IVC together for the in vitro production of bovine embryos [22].

The positive effect of melatonin was also evident in developmental kinetics, which is an important indicator of embryonic quality [45]. The faster embryonic development observed in the treated groups may be attributed to the combined effect of melatonin in protecting against oxidative stress, modulating cell cycle progression, and regulating apoptosis. These mechanisms collectively contribute to a more favorable environment for timely embryonic development, resulting in higher-quality embryos, as evidenced by their accelerated developmental kinetics. This is further supported by the reduced ROS levels observed in the melatonin-treated groups, reinforcing its protective role. Consistent with our findings, previous studies have reported similar reductions in ROS after using melatonin both in IVM and CIV of cattle [22,26,32,46], attributed to both its direct free radical scavenging properties and its ability to upregulate endogenous antioxidant enzymes such as *SOD* and *CAT* [47]. These enzymes play a crucial role in maintaining redox homeostasis within cells, further supporting embryo viability and development [48]. Despite the benefits in embryo production and kinetics, there was no difference in the total number of cells between the treatments with or without melatonin in the culture media. This suggests that melatonin may have beneficial effects on the quality of embryonic development and protect against oxidative stress without directly affecting cell numbers, which is in line with the findings of other studies in cattle using melatonin in IVM and IVC [22].

Due to the observed antioxidant effects of melatonin, we further analyzed the expression of genes related to oxidative stress to better understand how melatonin exerts its effects. In our study, when all treatments were compared, only the IMV + Mlt group showed higher levels of transcripts for *GSS* genes compared to the IVC + Mlt group. However, when each treatment was individually compared with the control, embryos from the IVC + Mlt group exhibited lower transcript levels of these oxidative stress-related genes. This may reflect a reduced need for endogenous antioxidant responses owing to the direct protective effect of melatonin. Such a response suggests a more stable redox environment in which embryos rely on pre-existing antioxidant proteins rather than upregulating gene transcription, indicating less stressful and more favorable conditions for embryonic development. These molecular findings support our overall results, indicating that on D7 of in vitro culture, the IVC + Mlt group not only showed superior developmental performance compared to the other melatonin-treated groups but was also the only group to exhibit significant molecular differences relative to the control. Collectively, these observations support the idea that melatonin contributes to a more stable and supportive environment for embryonic development, particularly when added during the IVC phase.

As melatonin has recently been reported to regulate lipid metabolism in porcine oocytes [28,29], we investigated its effect on lipid metabolism in bovine embryos. We found that melatonin reduced the accumulation of lipid droplets in bovine embryos in all groups. This effect may be attributed to melatonin-mediated modulation of key metabolic enzymes, lipid utilization promotion, and reduction in storage by increasing lipolysis and fatty acid β-oxidation [28,29]. Interestingly, we found that this lipid regulation effect was more pronounced with continued exposure to melatonin during IVM and IVC, reinforcing the link between oocyte lipid content and embryonic lipid stores [24]. To gain deeper insight into the molecular mechanisms underlying this phenotypic reduction, we analyzed the expression of key genes involved in lipid metabolism (*CPT1A*, *PLIN2*, and *PPARγ*). However, despite the observed decrease in lipid droplets, we did not detect any significant changes in the expression of these genes. This discrepancy may be due to effects on other metabolic pathways not evaluated here, such as AMPK or SIRT1 [49,50]. Overall, these findings suggest that the influence of melatonin on lipid content likely involves regulatory mechanisms beyond the direct regulation of gene expression.

Lipids are a primary source of energy through β-oxidation in mitochondria, which motivated the investigation of mitochondrial activity. The control group showed lower mitochondrial activity than the melatonin-treated groups. The reduction in intracellular lipid stores suggests greater oxidation of fatty acids by the mitochondria for ATP generation. Previous studies associate this lower lipid accumulation with increased mitochondrial β-oxidation, energy production, and better embryonic development [28,29,51]. However, when melatonin was used only in the IVC, mitochondrial activity was lower than that in the other treatment groups. Melatonin regulates the mitochondrial activity in porcine oocytes during maturation [30] and promotes mitochondrial biogenesis by activating specific genes [52]. Thus, it is possible that melatonin exposure during IVM may induce early mitochondrial adaptations, explaining the lower mitochondrial activity in the group supplemented with melatonin only during IVC.

We also evaluated whether melatonin supplementation improves the cryotolerance of embryos produced in vitro. Despite its beneficial effects on oxidative stress and embryonic development, no significant differences were observed between groups in terms of apoptosis rates or survival after cryopreservation. These findings suggest that although melatonin improves certain aspects of embryo quality, it does not directly improve resistance to vitrification and heating. This result was unexpected, as melatonin phenotypically reduced lipid droplet accumulation, an effect usually associated with improved cryotolerance. However, this lack of effect can be attributed to the multifactorial nature of cryotolerance, which is influenced by not only oxidative stress but also factors such as cell compaction, differentiation, genomic integrity, cytoplasmic organization, and membrane stability, which may not have been sufficiently modified by melatonin supplementation [53].

In addition to its antioxidant and metabolic roles, melatonin has recently been implicated in epigenetic reprogramming, which is crucial for proper embryonic development [30,32,54,55]. Therefore, we investigated whether melatonin supplementation influenced epigenetic events in bovine embryos. First, we quantified the mRNA levels of the key enzymes involved in DNA methylation and demethylation. By comparing each treatment with the control group, we observed that the expression levels of *TET1* and *TET3* were altered in the IVC + Mlt group. Specifically, this group showed higher mRNA levels of *TET3* and reduced *TET1* expression, indicating that melatonin is involved in epigenetic reprogramming in a stage-specific manner.

Studies suggest that *TET1* is typically expressed in early stages, such as in 2–16 cell embryos and in pluripotent cells. Its absence leads to increased promoter methylation and loss of the pluripotent state [56,57,58,59]. In contrast, TET3 is known to be crucial for the active demethylation of paternal DNA shortly after fertilization and is also related to the activation of pluripotent genes such as OCT4 and NANOG and genes related to preimplantation. Therefore, the expression of TET3 until preimplantation is crucial for embryonic maintenance and viability [58]. Thus, the observed profile of higher expression of TET3 transcripts and lower TET1 transcripts in embryos treated with melatonin during culture is in accordance with the expected regulation for the expanded blastocyst stage, reflecting a more appropriate epigenetic pattern compatible with healthy embryonic development. In addition, the IVM/IVC + Mlt group showed a significant increase in *DNMT3A* expression, which may indicate a potentially active resumption of de novo methylation, necessary for the stabilization of epigenetic profiles after demethylation [60]. To complement the gene expression data and gain deeper insights into the epigenetic impact of melatonin, we assessed global DNA methylation patterns by analyzing two representative repetitive elements, *Satellite I* and *LINE-1*.

We used these two specific repetitive DNAs, *Satellite I* and *LINE-1*, which are highly repetitive and pervasive DNA sequences in the genome, constitute an expressive part of the bovine genome, and are predominantly located in the centromeric and pericentromeric regions [2,61]. Therefore, these genes may reflect specific DNA methylation patterns throughout the genome [37,61,62,63,64].

In our study, the only difference detected was that the IVM + Mlt group showed lower methylation levels in the *LINE-1* region than the control group, suggesting that exposure to melatonin during IVM may be involved in the epigenetic regulation of repetitive elements. A similar pattern was observed in *Satellite I* of IVEP-derived blastocysts, which also exhibited [35,65].

The percentage of methylation of each CpG site in *Satellite I* was evaluated; the IVC + Mlt group showed a higher number of CpGs (9) that were less methylated compared to the control, indicating that melatonin may also specifically affect genomic methylation during embryo culture. Satellite sequences play a role in chromatin organization and gene regulation during early development, and their hypomethylation has been associated with chromatin decondensation and activation of embryonic genome transcription. Therefore, reduced methylation in these regions may contribute to better embryonic development. These findings corroborate those of previous studies demonstrating the influence of melatonin on epigenetic regulation in porcine embryos by interfering with both DNA methylation and gene expression profiles [30,31]. In the analysis of hyper/hypomethylation of the alleles, we observed that the IVC + Mlt group showed more hypomethylated alleles in *Satellite I* than the, corroborating the findings of the other studies mentioned above.

Methylation analysis of *Satellite I* and *LINE-1* revealed some specific hypomethylated CpG sites (DMCs) within these repetitive elements in the IVC + Mlt group compared to the control. Although these changes do not characterize a differentially methylated region (DMR), some sequences surrounding these DMCs show high similarity to known transcription factor-binding motifs, particularly those recognized by PAX family proteins. The presence of these motifs suggests that such regions may be potential binding sites for PAX transcription factors whose ability to bind to DNA is influenced by the methylation status of the target sequence. Members of the PAX gene family, which contain the conserved paired box domain, are key regulators of embryonic development and organogenesis, including the formation of muscle, kidney, neural crest, and eye [66,67,68,69,70,71]. In addition to their classical role in DNA binding and transcriptional regulation, PAX proteins can recruit chromatin remodeling complexes that modulate the chromatin landscape through activating or repressive histone marks [72,73,74,75]. Therefore, the observed hypomethylation may modulate the accessibility or binding affinity of PAX factors to these regions, potentially influencing downstream gene regulation; however, this hypothesis was not explored further in the present study.

Taken together, our findings suggest that the effect of melatonin on epigenetic markers is dependent on the stage at which it is added to the system, reinforcing the importance of the timing of melatonin supplementation during in vitro embryo production. Notably, in the IVC + Mlt group, there was an increase in *TET3* expression and a decrease in *DNMT3A* expression, which are enzymes involved in DNA demethylation and methylation, respectively, accompanied by a higher proportion of hypomethylated CpGs and alleles in the *Satellite I* region. These findings highlight that the IVC phase is a critical window for the epigenetic modulation mediated by melatonin.

Although the results presented are promising, some limitations must be acknowledged. The use of oocytes obtained from slaughterhouse-derived ovaries was necessary due to the high number of oocytes required to conduct the entire study, which may introduce uncontrolled biological variability. In addition, the gene expression analysis was restricted to a specific panel of genes related to oxidative stress, lipid metabolism, and epigenetic regulation, limiting a broader understanding of the molecular pathways involved. Finally, no in vivo evaluations or validations were performed. Therefore, future studies employing more specific and comprehensive approaches are necessary to confirm and expand the findings reported here.

## 5. Conclusions

This study provided novel evidence that melatonin positively regulates lipid and mitochondrial metabolism, a mechanism that has not yet been elucidated in cattle. It also elucidates the role of melatonin in epigenetic mechanisms that have not been elucidated in cattle. Among the melatonin-supplemented treatments, the IVC + Mlt treatment exhibited the most pronounced and consistent improvements. Collectively, these findings suggest that the addition of melatonin in vitro cultures is the most effective strategy for promoting the generation of embryos with superior developmental competence. Therefore, its use to optimize outcomes in bovine reproduction warrants further exploration and application in both research and commercial settings.

## Figures and Tables

**Table 1:** Primer information used for gene expression quantification by qPCR, including GenBank accession number, primer sequences, amplicon size (bp), melting temperature (Tm), primer efficiency, and average Ct value.

Genes	Sequences	[ ] Primer (nM)	AS (bp)	GB	PE (%)	MT (°C)	CT
*DNMT3A*	F: TTTCCAATGTGCCATGACAGCGAC R: GGGCCCACTCGATCATTTGTTTGT	200	82	NM_001206502.1	114.726	83	31.81
*DNMT3B*	F: CAACAAGCAACCAGAGAATAAG R: CAACATCCGAAGCCATTTG	200	161	NM_181813.2	112.048	85	34.60
*TET1*	F: GTATGCTCCAGCTGCTTATC R: CCACTGTGCTCCCATTATTC	200	167	XM_015469834.1	108.166	84	30.81
*TET 2*	F: GTAGGGACATTTCCTCCTTATTC R: CAGCTGCACTGTAGTTATGG	200	157	XM_010828077.2	105.302	81	34.73
*TET 3*	F: GTAACCCAGGTGATTCTGATAC R: CAGCAGCCTATCTGCTAATC	200	200	XM_015465317.1	101.853	81	34.39
*SOD 1*	F- GGGAGATACAGTCGTGGTAA R- CCAACATGCCTCTCTTCATC	300	171	NM_174615.2	105.63	82	30.90
*CAT*	F: GAATGAGGAGCAGAGGAAAC R: CTCCGACCCTCAGAGATTAG	300	241	NM_001035386.2	95.38	83	31.53
*GSS*	F- GAGAGGGTGGAGGTAACAA R- TCTTTCCCTCCCTGACATAG	300	213	NM_001015630.1	104.03	85	32.35
*CPT1A*	F- GTTGCTGATGACGGCTATG R- CCCAGAAGTGCTAAGAGATTTAC	300	199	NM_001304989.2	101.59	83	31.24
*PLIN2*	F: CGGCTACGATGATACAGATG R: TGCGAAACACAGAGTAGATG	300	200	NM_173980.2	93.63	85	31.88
*PPAR* *γ*	F- GTCAGTACTGTCGGTTTCAG R- CAGCGGGAAGGACTTTATG	300	200	NM_181024.2	100.152	85	34.77
*GAPDH*	F: GGCGTGAACCACGAGAAGTATAA R: CCCTCCACGATGCCAAAGT	300	118	NM_001034034.2	94.54	84	28.35

AS: amplicon size; GB: GenBank; PE: primer efficiency; F: forward; R: reverse; bp: base pair; MT: melting temperature.

**Table 2:** Primers for methylation analysis of *Satellite I* and *LINE-1* genes.

Genomic Region	Primer Sequence (5′-3′)	GenBank	CpG Sites	Amplicon Length (bp)
*Satellite I*	F: TGTAGATTGGGGATAGGAGAGTTAG R: CCCCTACTTTATCTAAAAAAAATTACCTT	AH001157.2	23	347
*LINE-1*	F: GGTTAATATTTGTTTGAGAAGGTG R: RTTTCCCTCTATTATATCTTCTTCTATTTAC	DQ000238.1	27	505

F (forward); R (reverse); bp (base pair).

**Table 3:** In vitro production of bovine embryos with different treatments using melatonin in the media.

Treatments	N. Oocytes	Cleavage (D2)	Blastocyst (D6)	Blastocyst (D7)
Control	1356	1132 (83.48%)	102 (7.52%)	481 (35.47%) ^a^
IVM + Mlt	1540	1295 (84.09%)	124 (8.05%)	659 (42.79%) ^bc^
IVC + Mlt	1533	1299 (84.74%)	114 (7.44%)	677 (44.16%) ^c^
IVM/IVC + Mlt	1377	1150 (83.51%)	108 (8.84%)	556 (40.38%) ^b^

Control group: no melatonin addition N: number; D2: day two; D6: day six; D7: day seven; Mlt: melatonin; IVM: in vitro maturation; IVC: in vitro culture. ^a,b,c^ Values with different letters within the same column are significantly different (*p* < 0.05).

**Table 4:** Embryonic development at 144 h (D6) and 168 h (D7) post-insemination under different melatonin treatments during in vitro. embryo production.

Treatments	Blastocyst (D6)					Blastocyst (D7)				
	EB	BL	BX	HB	Total	EB	BL	BX	HB	Total
Control	79 (77.5%) ^a^	22 (21.6%) ^a^	1 (0.9%) ^a^	0 (0.0%)	102	63 (13.1%)	139 (28.9%)	272 (56.5%)	7 (1.5%) ^a^	481
IVM + Mlt	72 (58.0%) ^b^	47 (37.9%) ^b^	5 (4.1%) ^ab^	0 (0.0%)	124	84 (12.7%)	195 (29.5%)	357 (54.2%)	23 (3.6%) ^b^	659
IVC + Mlt	69 (60.5%) ^b^	38 (33.3%) ^b^	7 (6.2%) ^b^	0 (0.0%)	114	82 (12.1%)	180 (26.6%)	392 (57.9%)	23 (3.4%) ^b^	677
IVM/IVC + Mlt	60 (55.5%) ^b^	43 (39.8%) ^b^	5 (4.7%) ^ab^	0 (0.0%)	108	72 (12.9%)	146 (26.2%)	309 (55.6%)	29 (5.3%) ^b^	556

Control group: No melatonin treatment N, number; D2: day two; D6: day six; D7: day seven; Mlt, melatonin; IVM, in vitro maturation; IVC, in vitro culture; EB: Early blastocyst; BL, blastocyst; BX, expanded blastocyst; HB, hatched blastocyst. ^a,b^ Different superscript letters within columns indicate significant differences (*p* < 0.05), as determined using the chi-square test.

**Table 5:** Rates of expanded blastocyst, hatched blastocyst, degenerated, non-re-expanded, and re-expanded embryos at 12 and 24 h post-warming.

Treatments	Warmed	12h Post-Warming					24h Post-Warming				
		BX	HD	DEG	NR	Re-Exp	BX	HB	DEG	NR	Re-Exp
Control	49	32 (65.31%)	5 (10.20%)	3 (6.12%)	9 (18.37%)	37 (75.51%)	23 (46.94%)	14 (28.57%)	3 (6.12%)	9 (18.37%)	37 (75.51%)
IVM + Mlt	53	35 (66.04%)	8 (15.09%)	4 (7.55%)	6 (11.32%)	43 (81.13%)	22 (41.51%)	21 (39.62%)	4 (7.55%)	6 (11.32%)	43 (81.13%)
IVC + Mlt	63	41 (65.08%)	11 (17.46%)	4 (6.35%)	7 (11.11%)	52 (82.54%)	25 (39.68%)	27 (42.86%)	4 (6.35%)	7 (11.11%)	52 (82.54%)
IVM/IVC + Mlt	51	33 (64.71%)	6 (11.76%)	4 (7.84%)	8 (15.69%)	39 (76.47%)	21 (41.18%)	18 (35.29%)	4 (7.84%)	8 (15.69%)	39 (76.47%)

Control group: no melatonin addition. Mlt: melatonin; IVM: in vitro maturation; IVC: in vitro culture; BX: expanded blastocyst; HB: hatched blastocyst; DEG: degenerated; NR: non-re-expanded; Re-Exp: Re-Expanded.

**Table 6:** Total cell number, number of apoptotic cells, and proportion of apoptotic cells in expanded blastocysts at 24 h post-warming.

Treatments	N. Embryos	Total Cell Number	Total Apoptotic Cell	Apoptosis Rate (%)
Fresh Control	22	186.50 ± 14.41	14.09 ± 3.22	7.60 ± 1.83
Control	24	181.67 ± 17.37	13.75 ± 3.35	7.64 ± 1.96
IVM + Mlt	20	181.50 ± 9.12	13.90 ± 3.51	7.72 ± 2.15
IVC + Mlt	19	185.84 ± 15.21	13.05 ± 3.91	7.06 ± 2.13
IVM/IVC + Mlt	21	179.48 ± 15.62	12.95 ± 3.73	7.19 ± 1.91

Control group: no melatonin addition. Mlt: melatonin; IVM: in vitro maturation; IVC: in vitro culture; N: number.

## Data Availability

All data generated or analyzed during this study are presented in the article. Raw datasets (e.g., images and numerical data) are available from the corresponding author upon reasonable request.

## Abbreviations

The following abbreviations are used in this manuscript:

IVEP	In vitro embryo production
IVM	In vitro maturation
IVC	In vitro culture
ROS	Reactive oxygen species
Mlt	Melatonin
SCNT	Somatic cell nuclear transfer
CT	Control
COCs	Cumulus-oocyte complexes
IVF	In vitro fertilization
SOF	Synthetic oviductal fluid
FBS	Fetal bovine serum
BSA	Bovine serum albumin

## References

[1] Rizos D., Ward F., Duffy P., Boland M.P., Lonergan P. (2002). Consequences of Bovine Oocyte Maturation, Fertilization or Early Embryo Development in Vitro versus in Vivo: Implications for Blastocyst Yield and Blastocyst Quality. Mol. Reprod. Dev..

[2] Holm P., Callesen H. (1998). In Vivo versus in Vitro Produced Bovine Ova: Similarities and Differences Relevant for Practical Application. Reprod. Nutr. Dev..

[3] Mo L., Ma J., Xiong Y., Xiong X., Lan D., Li J., Yin S. (2023). Factors Influencing the Maturation and Developmental Competence of Yak (Bos Grunniens) Oocytes In Vitro. Genes.

[4] Salek F., Guest A., Johnson C., Kastelic J.P., Thundathil J. (2025). Factors Affecting the Success of Ovum Pick-Up, In Vitro Production and Cryopreservation of Embryos in Cattle. Animals.

[5] Agarwal A., Aponte-Mellado A., Premkumar B.J., Shaman A., Gupta S. (2012). The Effects of Oxidative Stress on Female Reproduction: A Review. Reprod. Biol. Endocrinol..

[6] Agarwal A., Gupta S., Sekhon L., Shah R. (2008). Redox Considerations in Female Reproductive Function and Assisted Reproduction: From Molecular Mechanisms to Health Implications. Antioxid. Redox Signal..

[7] Agarwal A., Gupta S., Sharma R.K. (2005). Role of Oxidative Stress in Female Reproduction. Reprod. Biol. Endocrinol..

[8] Gutteridge J.M.C., Halliwell B. (2000). Free Radicals and Antioxidants in the Year 2000: A Historical Look to the Future. Ann. N. Y. Acad. Sci..

[9] Guemra S., Monzani P.S., Santos E.S., Zanin R., Ohashi O.M., Miranda M.S., Adona P.R. (2013). Maturação in Vitro de Oócitos Bovinos Em Meios Suplementados Com Quercetina e Seu Efeito Sobre o Desenvolvimento Embrionário. Arq. Bras. Med. Vet. Zootec..

[10] Guimarães A.L.S., Pereira S.A., Diógenes M.N., Dode M.A.N. (2016). Effect of Insulin–Transferrin–Selenium (ITS) and L-Ascorbic Acid (AA) during in Vitro Maturation on in Vitro Bovine Embryo Development. Zygote.

[11] Lee S., Jin J.-X., Taweechaipaisankul A., Kim G.A., Lee B.C. (2018). Synergistic Effects of Resveratrol and Melatonin on in Vitro Maturation of Porcine Oocytes and Subsequent Embryo Development. Theriogenology.

[12] An L., Liu J., Du Y., Liu Z., Zhang F., Liu Y., Zhu X., Ling P., Chang S., Hu Y. (2018). Synergistic Effect of Cysteamine, Leukemia Inhibitory Factor, and Y27632 on Goat Oocyte Maturation and Embryo Development in Vitro. Theriogenology.

[13] Magata F., Ideta A., Matsuda F., Urakawa M., Oono Y. (2021). Glutathione Ethyl Ester Improved the Age-Induced Decline in the Developmental Competence of Bovine Oocytes. Theriogenology.

[14] Cruz M.H.C., Leal C.L.V., Cruz J.F.D., Tan D.-X., Reiter R.J. (2014). Role of Melatonin on Production and Preservation of Gametes and Embryos: A Brief Review. Anim. Reprod. Sci..

[15] Brzezinski A. (1997). Melatonin in Humans. N. Engl. J. Med..

[16] Pang S.F., Li L., Ayre E.A., Pang C.S., Lee P.P.N., Xu R.K., Chow P.H., Yu Z.H., Shiu S.Y.W. (1998). Neuroendocrinology of Melatonin in Reproduction: Recent Developments. J. Chem. Neuroanat..

[17] Barrett P., Bolborea M. (2012). Molecular Pathways Involved in Seasonal Body Weight and Reproductive Responses Governed by Melatonin. J. Pineal Res..

[18] Do L.T.K., Shibata Y., Taniguchi M., Nii M., Nguyen T.V., Tanihara F., Takagi M., Otoi T. (2015). Melatonin Supplementation During In Vitro Maturation and Development Supports the Development of Porcine Embryos. Reprod. Domest. Anim..

[19] Rodriguez-Osorio N., Kim I.J., Wang H., Kaya A., Memili E. (2007). Melatonin Increases Cleavage Rate of Porcine Preimplantation Embryos in Vitro. J. Pineal Res..

[20] Wang F., Tian X., Zhou Y., Tan D., Zhu S., Dai Y., Liu G. (2014). Melatonin Improves the Quality of In Vitro Produced (IVP) Bovine Embryos: Implications for Blastocyst Development, Cryotolerance, and Modifications of Relevant Gene Expression. PLoS ONE.

[21] Hao T., Xu X., Hao H., Du W., Pang Y., Zhao S., Zou H., Yang S., Zhu H., Yang Y. (2021). Melatonin Improves the Maturation and Developmental Ability of Bovine Oocytes by Up-Regulating GJA4 to Enhance Gap Junction Intercellular Communication. Reprod. Fertil. Dev..

[22] Marques T., Da Silva Santos E., Diesel T., Leme L., Martins C., Dode M., Alves B., Costa F., De Oliveira E., Gambarini M. (2018). Melatonin Reduces Apoptotic Cells, SOD 2 and HSPB 1 and Improves the in Vitro Production and Quality of Bovine Blastocysts. Reprod. Domest. Anim..

[23] Tian X., Wang F., He C., Zhang L., Tan D., Reiter R.J., Xu J., Ji P., Liu G. (2014). Beneficial Effects of Melatonin on Bovine Oocytes Maturation: A Mechanistic Approach. J. Pineal Res..

[24] Bahadori M.H., Ghasemian F., Ramezani M., Asgari Z. (2013). Melatonin Effect during Different Maturation Stages of Oocyte and Subsequent Embryo Development in Mice. Iran. J. Reprod. Med..

[25] Yang J., Guo S., Pan B., Qazi I.H., Qin J., Zang S., Han H., Meng Q., Zhou G. (2021). Melatonin Promotes in Vitro Maturation of Vitrified-Warmed Mouse GV Oocytes Potentially by Modulating MAD2 Protein Expression of SAC Component through MTRs. Cryobiology.

[26] Yang M., Tao J., Chai M., Wu H., Wang J., Li G., He C., Xie L., Ji P., Dai Y. (2017). Melatonin Improves the Quality of Inferior Bovine Oocytes and Promoted Their Subsequent IVF Embryo Development: Mechanisms and Results. Molecules.

[27] Castello P.R., Drechsel D.A., Patel M. (2007). Mitochondria Are a Major Source of Paraquat-Induced Reactive Oxygen Species Production in the Brain. J. Biol. Chem..

[28] Jin J., Lee S., Taweechaipaisankul A., Kim G.A., Lee B.C. (2017). Melatonin Regulates Lipid Metabolism in Porcine Oocytes. J. Pineal Res..

[29] Jin J.-X., Sun J.-T., Jiang C.-Q., Cui H.-D., Bian Y., Lee S., Zhang L., Lee B.C., Liu Z.-H. (2022). Melatonin Regulates Lipid Metabolism in Porcine Cumulus–Oocyte Complexes via the Melatonin Receptor 2. Antioxidants.

[30] Liang S., Jin Y.-X., Yuan B., Zhang J.-B., Kim N.-H. (2017). Melatonin Enhances the Developmental Competence of Porcine Somatic Cell Nuclear Transfer Embryos by Preventing DNA Damage Induced by Oxidative Stress. Sci. Rep..

[31] Qu J., Sun M., Wang X., Song X., He H., Huan Y. (2020). Melatonin Enhances the Development of Porcine Cloned Embryos by Improving DNA Methylation Reprogramming. Cell. Reprogramming.

[32] Su J., Wang Y., Xing X., Zhang L., Sun H., Zhang Y. (2015). Melatonin Significantly Improves the Developmental Competence of Bovine Somatic Cell Nuclear Transfer Embryos. J. Pineal Res..

[33] Machado G.M., Carvalho J.O., Filho E.S., Caixeta E.S., Franco M.M., Rumpf R., Dode M.A.N. (2009). Effect of Percoll Volume, Duration and Force of Centrifugation, on in Vitro Production and Sex Ratio of Bovine Embryos. Theriogenology.

[34] Holm P., Booth P.J., Schmidt M.H., Greve T., Callesen H. (1999). High Bovine Blastocyst Development in a Static in Vitro Production System Using Sofaa Medium Supplemented with Sodium Citrate and Myo-Inositol with or without Serum-Proteins. Theriogenology.

[35] Vandesompele J., De Preter K., Pattyn F., Poppe B., Van Roy N., De Paepe A., Speleman F. (2002). Accurate Normalization of Real-Time Quantitative RT-PCR Data by Geometric Averaging of Multiple Internal Control Genes. Genome Biol..

[36] Pfaffl M.W. (2001). A New Mathematical Model for Relative Quantification in Real-Time RT-PCR. Nucleic Acids Res..

[37] López-Flores I., Garrido-Ramos M.A., Garrido-Ramos M.A. (2012). The Repetitive DNA Content of Eukaryotic Genomes. Genome Dynamics.

[38] Mendonça A.D.S., Guimarães A.L.S., Silva N.M.A.D., Caetano A.R., Dode M.A.N., Franco M.M. (2015). Characterization of the IGF2 Imprinted Gene Methylation Status in Bovine Oocytes during Folliculogenesis. PLoS ONE.

[39] Kumaki Y., Oda M., Okano M. (2008). QUMA: Quantification Tool for Methylation Analysis. Nucleic Acids Res..

[40] Kuwayama M., Vajta G., Kato O., Leibo S.P. (2005). Highly Efficient Vitrification Method for Cryopreservation of Human Oocytes. Reprod. Biomed. Online.

[41] Fidelis A.A.G., De Oliveira Fernandes G., Melo F.R., Leme L.D.O., Adona P.R., Kawamoto T.S., Dode M.A.N. (2020). Ethanolic Extract of Dried Leaves from the Cerrado Biome Increases the Cryotolerance of Bovine Embryos Produced In Vitro. Oxidative Med. Cell. Longev..

[42] Wang S., Liu B., Liu W., Xiao Y., Zhang H., Yang L. (2017). The Effects of Melatonin on Bovine Uniparental Embryos Development in Vitro and the Hormone Secretion of COCs. PeerJ.

[43] Zhao X., Wang N., Hao H., Li C., Zhao Y., Yan C., Wang H., Du W., Wang D., Liu Y. (2018). Melatonin Improves the Fertilization Capacity and Developmental Ability of Bovine Oocytes by Regulating Cytoplasmic Maturation Events. J. Pineal Res..

[44] Lira A.D.S., Chaves R.D.M., Moraes Junior F.D.J., Costa Junior S.H., Amaral B.K.L.D., Trovão H.M.P. (2020). Use of Melatonin in the In Vitro Production of Bovine Embryos. Rev. Bras. Saúde Prod. Anim..

[45] Huayhua C., Rodríguez M., Vega J., Briones M., Rodriguez-Alvarez L., Mellisho E. (2023). Blastulation Time Measured with Time-Lapse System Can Predict in Vitro Viability of Bovine Blastocysts. PLoS ONE.

[46] Gao C., Han H., Tian X., Tan D., Wang L., Zhou G., Zhu S., Liu G. (2012). Melatonin Promotes Embryonic Development and Reduces Reactive Oxygen Species in Vitrified Mouse 2-cell Embryos. J. Pineal Res..

[47] Cavallari F.D.C., Leal C.L.V., Zvi R., Hansen P.J. (2019). Effects of Melatonin on Production of Reactive Oxygen Species and Developmental Competence of Bovine Oocytes Exposed to Heat Shock and Oxidative Stress during in Vitro Maturation. Zygote.

[48] Rodriguez C., Mayo J.C., Sainz R.M., Antolín I., Herrera F., Martín V., Reiter R.J. (2004). Regulation of Antioxidant Enzymes: A Significant Role for Melatonin. J. Pineal Res..

[49] Ahmadi Z., Ashrafizadeh M. (2020). Melatonin as a Potential Modulator of Nrf2. Fundam. Clin. Pharmacol..

[50] Li Q., Tang Y., Chen Y., Li B., Wang H., Liu S., Adeniran S.O., Zheng P. (2024). Melatonin Regulates the Expression of VEGF and HOXA10 in Bovine Endometrial Epithelial Cells through the SIRT1/PI3K/AKT Pathway. Animals.

[51] Vasconcelos E.M., Braga R.F., Leal G.R., Carvalho R.P.R., Machado-Neves M., Sudano M.J., Souza-Fabjan J.M.G. (2024). Impact of Reducing Lipid Content during in Vitro Embryo Production: A Systematic Review and Meta-Analysis. Theriogenology.

[52] Izyumov D.S., Domnina L.V., Nepryakhina O.K., Avetisyan A.V., Golyshev S.A., Ivanova O.Y., Korotetskaya M.V., Lyamzaev K.G., Pletjushkina O.Y., Popova E.N. (2010). Mitochondria as Source of Reactive Oxygen Species under Oxidative Stress. Study with Novel Mitochondria-Targeted Antioxidants—the “Skulachev-Ion” Derivatives. Biochem. Mosc..

[53] Marques T.C., Santos E.C.D.S., Diesel T.O., Martins C.F., Cumpa H.C.B., Leme L.D.O., Dode M.A.N., Alves B.G., Costa F.P.H., Oliveira E.B.D. (2021). Blastocoel Fluid Removal and Melatonin Supplementation in the Culture Medium Improve the Viability of Vitrified Bovine Embryos. Theriogenology.

[54] Korkmaz A., Reiter R.J. (2008). Epigenetic Regulation: A New Research Area for Melatonin?. J. Pineal Res..

[55] Tutt D.A.R., Guven-Ates G., Kwong W.Y., Simmons R., Sang F., Silvestri G., Canedo-Ribeiro C., Handyside A.H., Labrecque R., Sirard M.-A. (2023). Developmental, Cytogenetic and Epigenetic Consequences of Removing Complex Proteins and Adding Melatonin during in Vitro Maturation of Bovine Oocytes. Front. Endocrinol..

[56] Ito S., D’Alessio A.C., Taranova O.V., Hong K., Sowers L.C., Zhang Y. (2010). Role of Tet Proteins in 5mC to 5hmC Conversion, ES-Cell Self-Renewal and Inner Cell Mass Specification. Nature.

[57] Linowiecka K., Slominski A.T., Reiter R.J., Böhm M., Steinbrink K., Paus R., Kleszczyński K. (2023). Melatonin: A Potential Regulator of DNA Methylation. Antioxidants.

[58] Montgomery T., Uh K., Lee K. (2024). TET Enzyme Driven Epigenetic Reprogramming in Early Embryos and Its Implication on Long-Term Health. Front. Cell Dev. Biol..

[59] Zhang J., Hao L., Wei Q., Zhang S., Cheng H., Zhai Y., Jiang Y., An X., Li Z., Zhang X. (2020). TET3 Overexpression Facilitates DNA Reprogramming and Early Development of Bovine SCNT Embryos. Reproduction.

[60] Okano M., Bell D.W., Haber D.A., Li E. (1999). DNA Methyltransferases Dnmt3a and Dnmt3b Are Essential for de Novo Methylation and Mammalian Development. Cell.

[61] Silveira M.M., Salgado Bayão H.X., Dos Santos Mendonça A., Borges N.A., Vargas L.N., Caetano A.R., Rumpf R., Franco M.M. (2018). DNA Methylation Profile at a Satellite Region Is Associated with Aberrant Placentation in Cloned Calves. Placenta.

[62] Spadafora C. (2015). A LINE-1–Encoded Reverse Transcriptase–Dependent Regulatory Mechanism Is Active in Embryogenesis and Tumorigenesis. Ann. N. Y. Acad. Sci..

[63] De Oliveira Leme L., Franco M.M., De Faria O.A., Caetano A.R., De Souza J.G., De Rezende Carvalheira L., De Siqueira Filho E., Dode M.A.N. (2025). Reduction of Nutrients Concentration in Culture Medium Has No Effect on Bovine Embryo Production, Pregnancy and Birth Rates. Sci. Rep..

[64] Pezer Ž., Brajković J., Feliciello I., Ugarković Đ., Garrido-Ramos M.A. (2012). Satellite DNA-Mediated Effects on Genome Regulation. Genome Dynamics.

[65] Kaneda M., Akagi S., Watanabe S., Nagai T. (2011). Comparison of DNA Methylation Levels of Repetitive Loci during Bovine Development. BMC Proc..

[66] Blake J.A., Ziman M.R. (2014). Pax Genes: Regulators of Lineage Specification and Progenitor Cell Maintenance. Development.

[67] Dahl E., Koseki H., Balling R. (1997). *Pax* Genes and Organogenesis. BioEssays.

[68] Noll M. (1993). Evolution and Role of Pax Genes. Curr. Opin. Genet. Dev..

[69] Paixão-Côrtes V.R., Salzano F.M., Bortolini M.C. (2015). Origins and Evolvability of the PAX Family. Semin. Cell Dev. Biol..

[70] Shaw T., Barr F.G., Üren A. (2024). The PAX Genes: Roles in Development, Cancer, and Other Diseases. Cancers.

[71] Wang Q., Fang W., Krupinski J., Kumar S., Slevin M., Kumar P. (2008). *Pax* Genes in Embryogenesis and Oncogenesis. J. Cell. Mol. Med..

[72] Abraham S., Paknikar R., Bhumbra S., Luan D., Garg R., Dressler G.R., Patel S.R. (2015). The Groucho-Associated Phosphatase PPM1B Displaces Pax Transactivation Domain Interacting Protein (PTIP) to Switch the Transcription Factor Pax2 from a Transcriptional Activator to a Repressor. J. Biol. Chem..

[73] Kim D., Patel S.R., Xiao H., Dressler G.R. (2009). The Role of PTIP in Maintaining Embryonic Stem Cell Pluripotency. Stem Cells.

[74] Mayran A., Pelletier A., Drouin J. (2015). Pax Factors in Transcription and Epigenetic Remodelling. Semin. Cell Dev. Biol..

[75] Patrício P., Ramalho-Carvalho J., Costa-Pinheiro P., Almeida M., Barros-Silva J.D., Vieira J., Dias P.C., Lobo F., Oliveira J., Teixeira M.R. (2013). Deregulation of PAX 2 Expression in Renal Cell Tumours: Mechanisms and Potential Use in Differential Diagnosis. J. Cell. Mol. Med..

